# Intracellular NAD^+^ Depletion Confers a Priming Signal for NLRP3 Inflammasome Activation

**DOI:** 10.3389/fimmu.2021.765477

**Published:** 2021-12-20

**Authors:** Do-Wan Shim, Hyo-Joung Cho, Inhwa Hwang, Taek-Yeol Jung, Hyun-Seok Kim, Ju Hee Ryu, Je-Wook Yu

**Affiliations:** ^1^ Department of Microbiology and Immunology, Institute for Immunology and Immunological Diseases, Brain Korea 21 Project for Medical Science, Yonsei University College of Medicine, Seoul, South Korea; ^2^ Department of Life Science, College of Natural Science, The Research Center for Cellular Homeostasis, Ewha Womans University, Seoul, South Korea; ^3^ Theragnosis Research Center, Biomedical Research Division, Korea Institute of Science and Technology (KIST), Seoul, South Korea

**Keywords:** NAD, aging, macrophage, proinflammatory, inflammasome

## Abstract

Nicotinamide adenine dinucleotide (NAD^+^) is an important cofactor in many redox and non-redox NAD^+^-consuming enzyme reactions. Intracellular NAD^+^ level steadily declines with age, but its role in the innate immune potential of myeloid cells remains elusive. In this study, we explored whether NAD^+^ depletion by FK866, a highly specific inhibitor of the NAD salvage pathway, can affect pattern recognition receptor-mediated responses in macrophages. NAD^+^-depleted mouse bone marrow-derived macrophages (BMDMs) exhibited similar levels of proinflammatory cytokine production in response to LPS or poly (I:C) stimulation compared with untreated cells. Instead, FK866 facilitated robust caspase-1 activation in BMDMs in the presence of NLRP3-activating signals such as ATP and nigericin, a potassium ionophore. However, this FK866-mediated caspase-1 activation was completely abolished in *Nlrp3*-deficient macrophages. FK866 plus nigericin stimulation caused an NLRP3-dependent assembly of inflammasome complex. In contrast, restoration of NAD^+^ level by supplementation with nicotinamide mononucleotide abrogated the FK866-mediated caspase-1 cleavage. FK866 did not induce or increase the expression levels of NLRP3 and interleukin (IL)-1β but drove mitochondrial retrograde transport into the perinuclear region. FK866-nigericin-induced mitochondrial transport is critical for caspase-1 cleavage in macrophages. Consistent with the *in vitro* experiments, intradermal coinjection of FK866 and ATP resulted in robust IL-1β expression and caspase-1 activation in the skin of wild-type, but not *Nlrp3*-deficient mice. Collectively, our data suggest that NAD^+^ depletion provides a non-transcriptional priming signal for NLRP3 activation *via* mitochondrial perinuclear clustering, and aging-associated NAD^+^ decline can trigger NLRP3 inflammasome activation in ATP-rich environments.

## Introduction

Aging is a complex and multisystem process characterized by a decline in the physiological integrity of an organism, leading to tissue degeneration (1). Aging in itself is not a disease; however, aged tissues are more susceptible to multiple disease-causing risk factors (2). Aging is often accompanied by chronic low-grade inflammation, known as inflammaging (3). Inflammaging can be triggered by endogenous metabolites or cellular debris and contributes to the etiology of major aging-associated diseases (4–6). However, the molecular underpinnings of how aging-related changes promote or propagate inflammation need further investigation.

Intracellular nicotinamide adenine dinucleotide (NAD^+^) levels steadily decline with age in both rodents and humans (7). NAD^+^ is an essential electron acceptor in several redox reactions that maintain intracellular homeostasis (8). NAD^+^ also functions as a cofactor for non-redox NAD^+^-consuming enzymes, such as poly-ADP-ribose polymerases (PARPs) and sirtuins (SIRTs) (9). NAD^+^ is synthesized either from tryptophan in the *de novo* pathway or by recycling nicotinamide (NAM) in the salvage pathway (9). In mammals, the salvage pathway is the predominant source of NAD^+^ biosynthesis due to its high adaptability (7). Nicotinamide phosphoribosyltransferase (NAMPT), the rate-limiting enzyme for NAD^+^ biosynthesis in the salvage pathway, converts NAM to nicotinamide mononucleotide (NMN), which is subsequently converted into NAD^+^ by NMN adenyltransferase (10). Reduced NAMPT expression at both mRNA and protein levels has been observed in multiple tissues during aging and is primarily responsible for the aging-associated NAD^+^ decline (11–13).

NAD^+^ decline is implicated in the pathophysiology of various diseases, including metabolic, cardiovascular, and neurodegenerative diseases (14). The supplementation of NAD^+^ using NAD^+^ pathway intermediates attenuates these degenerative disorders (11). Thus, NAD^+^ biosynthesis can be a potent therapeutic target for many aging-associated diseases. However, it is unclear whether NAD^+^ depletion can trigger or promote chronic proinflammatory responses that are closely associated with increased susceptibility to aging-associated diseases. Of note, a previous study showed that NAD^+^ depletion inhibits lipopolysaccharide (LPS)-induced Toll-like receptor (TLR) signaling in human monocytes (15). Similarly, inhibition of NAMPT (using FK866, a NAMPT-specific inhibitor) modulated the proinflammatory responses in macrophages (16). In this context, we assessed whether FK866-induced NAD^+^ decline can modulate pattern-recognition receptor (PRR)-mediated responses in myeloid cells. Consequently, we propose that NAD^+^ depletion can trigger NLRP3 activation in macrophages and induce *in vitro* and *in vivo* inflammasome activation in the presence of NLRP3-activating stimuli.

## Materials and Methods

### Mice

C57BL/6 (Orient Bio) and *Nlrp3* ^-/-^ (Jackson Laboratory) mice were bred at Yonsei University College of Medicine under specific pathogen-free conditions. To obtain myeloid-specific *Sirt1*-deficient mice (*Sirt1* ^fl/fl^;LysM Cre mice), homozygous *Sirt1* ^fl/fl^ mice (C57BL/6) were crossed with *LysM Cre* transgenic mice (C57BL/6, Jackson laboratory). Mice aged 9–12 weeks were used in the experiments. All experimental procedures were approved by the Institutional Ethical Committee, Yonsei University College of Medicine. Animal experiments were performed in accordance with the guidelines of the Institutional Ethical Committee. Mice were shaved 24 h prior to injection, and intradermally administered with FK866 (7 mg/kg) once a day, for two consecutive days. After the last FK866 injection, ATP was intradermally administered (12.5 mg/kg) at the same injection site. Six hours after ATP injection, the mice were sacrificed and subjected to various analyses.

### Reagents and Antibodies

FK866, lipopolysaccharide (LPS), nigericin, ATP, poly (dA:dT), poly (I:C) and nicotinamide mononucleotide (NMN) were obtained from Sigma-Aldrich (St. Louis, MO, USA). Flagellin purified from *P. aeruginosa* was obtained from *In vivo*Gen (San Diego, CA, USA). FK866 used in the *in vivo* experiments was purchased from Cayman (Ann arbor, MI, USA). Ciliobrevin D was obtained from Calbiochem (San Diego, CA, USA). Anti-mouse caspase-1 and anti-mouse NLRP3 antibodies were purchased from Adipogen (San Diego, CA, USA). Anti-apoptosis-associated speck-like protein containing a caspase recruitment domain (ASC) antibody was purchased from Cell Signaling Technology (Beverly, MA, USA). Anti-mouse IL-1β antibody was obtained from R&D Systems (Minneapolis, MN, USA). Anti-mouse gasdermin D (GSDMD) and anti-VDAC1 antibodies were purchased from Abcam (Cambridge, MA, USA). Anti-mouse β-actin antibody was purchased from Santa Cruz Biotechnology (Santa Cruz, CA, USA).

### Cell Culture

Mouse bone marrow cells were isolated from the femurs of C57BL/6, *Nlrp3* ^-/-^ or *Sirt1* ^-/-^ mice and cultured in L929-conditioned DMEM for 5–7 days to differentiate them into bone marrow-derived macrophages (BMDMs). BMDMs were maintained in L929-conditioned DMEM supplemented with 10% fetal bovine serum, and antibiotics. Immortalized NLRP3-GFP-expressing BMDMs were provided by Dr. E.S. Alnemri (Thomas Jefferson University, Philadelphia, USA).

### Intracellular NAD^+^ Quantification

Intracellular NAD^+^ level was measured by NAD/NADH-Glo™ Assay kit (Promega, WI, USA), according to the manufacturer’s instructions. Briefly, cells were grown in a 96-well plate. After appropriate treatment, cells were washed and lysed in bicarbonate buffer containing 1% dodecyl trimethyl ammonium bromide, followed by treatment with 0.4 N HCl at 60°C for 15 min. After neutralization with 0.5 M Trizma base, NAD/NADH-Glo™ Detection Reagent was added to each sample. The plates were incubated at room temperature for 30 min, and the luminescence was recorded using a microplate luminometer (Centro XS3 LB960, Berthold).

### Assay of PRR-Mediated Response

To activate TLR or RIG-I-like receptor (RLR) signaling, cells were treated with LPS or transfected with poly (I:C), respectively. TLR- or RLR-mediated responses were determined by measuring the extracellular secretion of IL-6 or TNF-α using a Quantikine ELISA Kit (R&D Systems, Minneapolis, MN, USA) or cellular mRNA expression of the cytokines using quantitative real-time PCR. To induce NLRP3 inflammasome activation, cells were treated with LPS, followed by treatment with ATP or nigericin. To stimulate AIM2 or NLRC4 inflammasome, cells were transfected with poly (dA:dT) or flagellin, respectively, using Lipofectamine 2000. Inflammasome activation was determined by the presence of active caspase-1 (p20) and active IL-1β (p17) in the culture supernatants using immunoblotting and by extracellular IL-1β quantification using ELISA.

### Assay of NLRP3 Inflammasome Assembly

To measure the oligomerization of NLRP3, speck-like aggregates of NLRP3-GFP were assessed using confocal microscopy, in NLRP3-GFP-expressing BMDMs. To determine the oligomerization of ASC, discuccinimidyl suberate (DSS, Thermo Scientific)-mediated cross-linking assay was performed as described previously (17).

### Immunoblot Analysis

Cells were lysed in a buffer containing 25 mM Tris-Cl (pH 7.5), 150 mM NaCl, 1% NP-40, 1% sodium deoxycholate, 0.1% SDS, and protease inhibitors. Soluble lysates were fractionated using SDS-polyacrylamide gel electrophoresis and transferred to polyvinylidene difluoride membranes. Cell culture supernatants were precipitated using a methanol/chloroform mixture as described previously (18) and immunoblotting was performed. All blot images are representative of at least three independent experiments.

### Cytokine mRNA Expression

Total RNA was extracted using TRIzol reagent (Invitrogen) and reverse transcribed using a PrimScript RT Master Mix (Takara) according to the manufacturers’ protocol. Template cDNA was amplified using SYBR Premix Ex Taq (TaKaRa) by quantitative real-time PCR. Primers used were as follows: 5′ - AGT TGC CTT CTT GGG ACT GA -3′ and 5′ - TCC ACG ATT TCC CAG AGA AC -3′ (mouse *Il-6*); 5′-GCC CAT CCT CTG TGA CTC AT-3′ and 5′-AGG CCA CAG GTA TTT TFT CG-3′ (mouse *Il-1β*); 5′-CGT CAG CCG ATT TGC TAT CT-3′ and 5’-CGT CAG CCG ATT TGC TAT CT-3’ (mouse *TNF-α*); 5′-CGC GGT TCT ATT TTG TTG GT-3′ and 5′-AGT CGG CAT CGT TTA TGG TC-3′ (mouse *Rn18s*).

### Immunofluorescence Assay

Cells were grown on coverslips in 12 or 24-well plates. Following treatment, cells were fixed using 4% formaldehyde and permeabilized using 0.2% Triton X-100. After blocking with 4% BSA, cells were incubated with anti-Tom 20 antibody (Cell signaling) and Phalloidin-Alexa488 (Invitrogen), followed by the Cy3-conjugated anti-rabbit IgG (Jackson Immuno Research or Invitrogen), and observed using a confocal microscope (Zeiss, LSM700 or LSM780). To quantify perinuclear mitochondria, the ratio of Cy3 fluorescence intensity of the region surrounding nucleus region (within 5 μm) to the total intracellular Cy3 fluorescence intensity was calculated using ZEN microscopy software.

### Detection of *In Vivo* Caspase-1 Activation

A Caspase-1-activatable probe was synthesized according to a previous study (19). To detect active caspase-1 in the skin of mice, caspase-1 probe (100 μg/100 μl of saline/mouse) was intravenously injected *via* tail 2 h before measurement. *In vivo* fluorescence in mouse skin was determined using an IVIS spectrum *In Vivo* imaging system (PerkinElmer, Waltham, MA, USA). The fluorescence intensity was analyzed using the Living Image software.

### Statistical Analysis

All values were expressed as the mean ± SEM. Data were analyzed using one-way analysis of variance (ANOVA) with Dunnett’s post-test for the comparison of all groups with control group or two-way ANOVA with Bonferroni post-test for comparisons between two groups. *p* values ≤ 0.05 were considered significant. Analyses were performed using GraphPad Prism 5.

## Results

### Intracellular NAD^+^ Depletion by FK866 Promotes Inflammasome Activation in the Presence of ATP or Nigericin Costimulation

**Figure 1:**
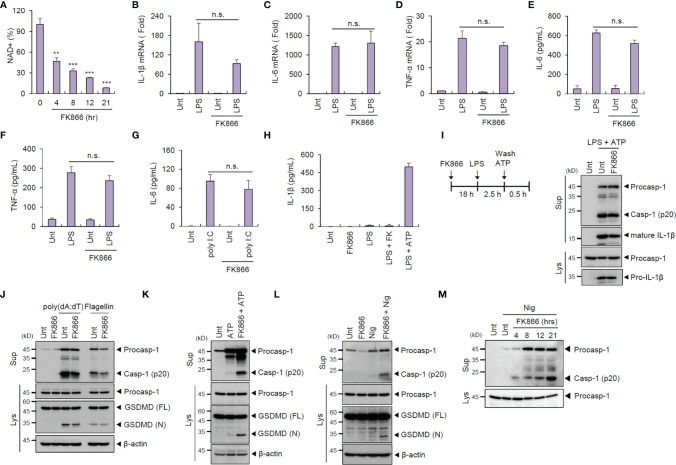
FK866-induced NAD^+^ depletion promotes caspase-1 activation in macrophages costimulated with ATP or nigericin. **(A)** Quantification of intracellular NAD^+^ level in mouse BMDMs treated with FK866 (100 nM) for the indicated times (*n* = 2). **(B–D)** Quantification of *Il-1β* **(B)**, *Il-6* **(C)**, or *Tnf-α* **(D)** mRNA levels in mouse BMDMs pretreated with FK866 (100 nM, 21 h), followed by LPS treatment (0.1 µg/ml, 3 h, *n* = 3). **(E–G)** Quantification of IL-6 or TNF-α in culture supernatants of mouse BMDMs treated with LPS (0.1 µg/ml, 3 h) or transfected with poly (I:C) (1 µg/ml, 3 h) in the presence or absence of FK866 pretreatment (*n* = 3). **(H)** Quantification of IL-1β in culture supernatants of mouse BMDMs treated with FK866 (100 nM, 4 h) or LPS (0.25 µg/ml, 2 h) alone, LPS followed by FK866 (4 h), or LPS followed by ATP (3 mM, 0.5 h) treatment (*n* = 4). **(I)** Immunoblots from mouse BMDMs treated with FK866 (21 h), followed by LPS and ATP (3 mM, 1 h) treatment. **(J)** Immunoblots from mouse BMDMs transfected with poly (dA:dT) (1 µg/ml, 2 h) or flagellin (0.25 µg/ml, 2 h) in the presence or absence of FK866 pretreatment. **(K, L)** Immunoblots from mouse BMDMs treated with ATP (3 mM, 1 h) or nigericin (5 μM, 1 h) in the presence or absence of FK866 pretreatment (100 nM, 21 h). **(M)** Immunoblots from mouse BMDMs treated with nigericin (5 μM, 1 h) in the presence of FK866 pretreatment (100 nM, 4~21 h). Cell culture supernatants (Sup) or cell lysates (Lys) were immunoblotted with the indicated antibodies. **P < 0.005, ***P < 0.001, n.s., not significant.

Mouse BMDMs treated with FK866 (100 nM, 21 h), a highly specific inhibitor of NAMPT, exhibited a significant reduction in intracellular NAD^+^ (**Figure 1A**) but without any cytotoxic effects (data not shown). We then examined the effect of NAD^+^ depletion on the innate immune response in macrophages against the following PRR ligands. FK866-induced NAD^+^ depletion did not impair or increase the LPS-triggered induction of proinflammatory cytokines such as IL-1β, IL-6, and TNF-α, as determined by their cellular mRNA levels in macrophages (**Figures 1B–D**). Subsequently, LPS-induced secretion of proinflammatory cytokines was not affected by FK866 (**Figures 1E, F**). FK866-pretreated BMDMs exhibited similar levels of poly (I:C)-triggered IL-6 production compared with untreated cells (**Figure 1G**). Meanwhile, FK866 pretreatment caused a slightly-increased production of type 1 interferon in response to LPS stimulation (**Supplementary Figure 1**). These data suggest that NAD^+^ depletion did not significantly impair both TLR4- and RLR-mediated responses in macrophages.

The effect of NAD^+^ decline on inflammasome signaling was then assessed. FK866 pretreatment regardless of LPS costimulation failed to induce IL-1β secretion by BMDMs (**Figure 1H**), suggesting that NAD^+^ depletion alone cannot trigger inflammasome activation. Additionally, FK866-pretreated BMDMs showed normal caspase-1 activation in response to NLRP3-activating stimulus (LPS + ATP) (**Figure 1I**). Similarly, NAD^+^ depletion did not affect both AIM2- and NLRC4-mediated inflammasome activation promoted by the transfection of poly (dA:dT) and flagellin, respectively (**Figure 1J**).

Next, we examined whether NAD^+^ depletion can act as an inflammasome-priming signal. Treatment with NLRP3-activating second signal alone, such as ATP and nigericin, did not induce caspase-1 activation but caused robust caspase-1 cleavage following FK866 pretreatment (**Figures 1K, L**). These NLRP3-activating second signals (ATP and nigericin) were thought to contribute to the NLRP3 activation *via* inducing potassium efflux (20). ATP or nigericin stimulation in NAD^+^-depleted BMDMs resulted in the cleavage of gasdermin D (GSDMD), a specific caspase-1 substrate (**Figures 1K, L**). Moreover, FK866 treatment induced a time-dependent caspase-1 activation in BMDMs (**Figure 1M**). These results indicate that NAD^+^ depletion followed by NLRP3-activating second signals can trigger inflammasome activation.

### NAD^+^ Depletion Promotes NLRP3-Dependent Inflammasome Activation in the Presence of an NLRP3 Stimulator

**Figure 2:**
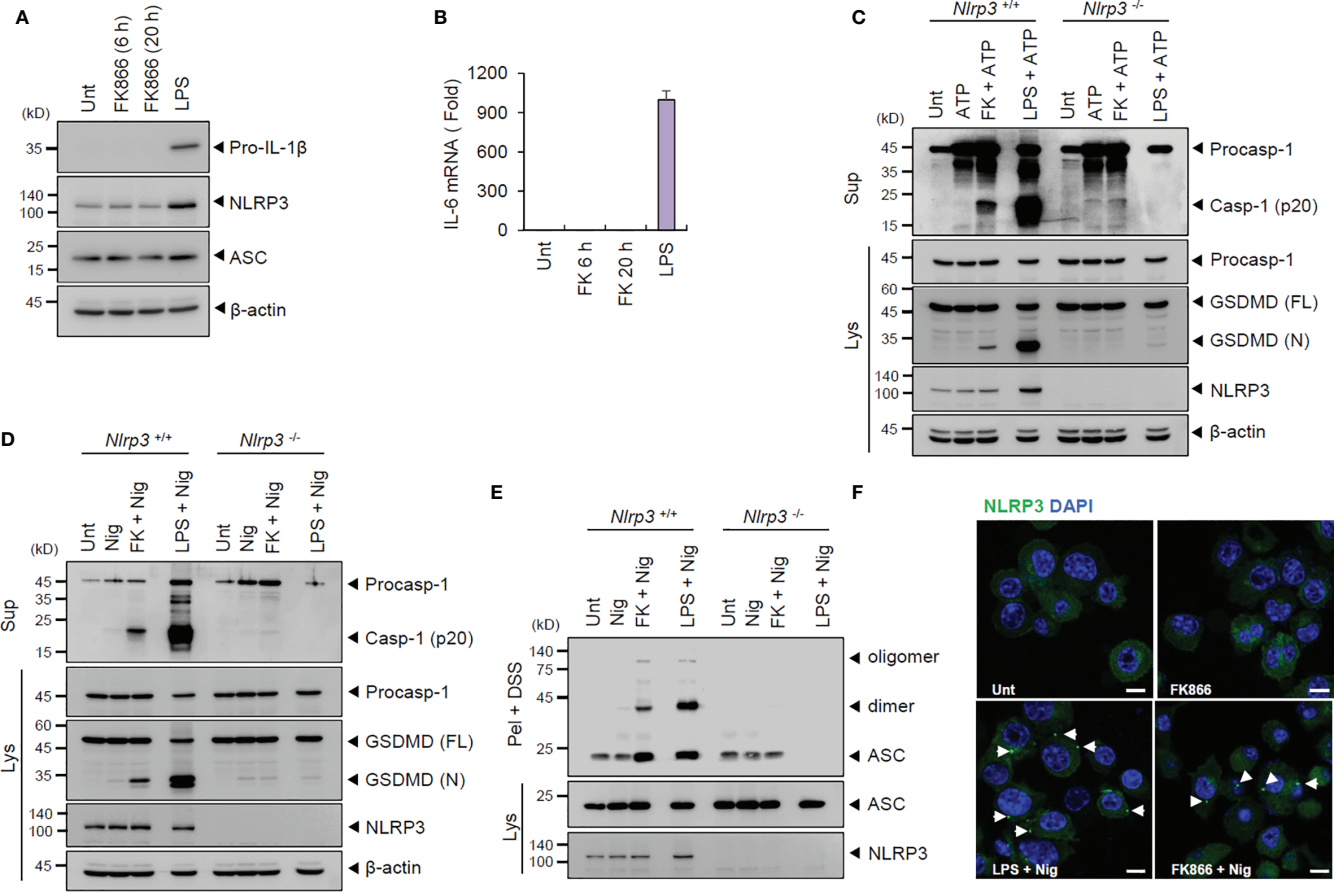
FK866-induced NAD^+^ depletion promotes NLRP3-mediated inflammasome activation in the presence of ATP or nigericin stimulation. **(A)** Immunoblots from the cell lysates of mouse BMDMs treated with FK866 (100 nM, 6 or 20 h) or LPS (0.1 µg/ml, 3 h). **(B)** Quantification of *Il-6* mRNA levels in mouse BMDMs treated as in **(A)**. **(C, D)** Immunoblots of *Nlrp3 ^+/+^ or Nlrp3 ^-/-^* mouse BMDMs treated with ATP (3 mM, 1 h) or nigericin (5 μM, 1 h) alone, or FK866 (100 nM, 21 h) or LPS (0.1 µg/ml, 3 h), followed by ATP or nigericin treatment. **(E)** Immunoblots of disuccinimidyl suberate (DSS)-crosslinked pellets (pel + DSS) or cellular lysates (Lys) from *Nlrp3^+/+^ or Nlrp3^-/-^* mouse BMDMs treated with FK866 or LPS, followed by treatment with nigericin. **(F)** Representative immunofluorescence images of NLRP3-GFP-expressing BMDMs treated with FK866 or LPS, followed by nigericin treatment. Arrows indicate speck-like aggregates of NLRP3 (green). DAPI represents the nuclear signal (blue). Scale bars, 10 μm. Cell culture supernatants (Sup) or cell lysates (Lys) were immunoblotted with the indicated antibodies.

To examine whether FK866 functions as a priming signal for NLRP3 activation, we assessed the induction of NLRP3 or proinflammatory cytokines in BMDMs. However, unlike LPS, FK866 did not upregulate the expression of inflammasome components, such as NLRP3, ASC and pro-IL-1β in BMDMs (**Figure 2A**). In addition, FK866 treatment failed to induce or increase IL-6 and P2X7 mRNA production (**Figure 2B** and **Supplementary Figure 2**). These findings suggest that FK866-induced NAD^+^ decline might act as a priming signal for NLRP3 activation in the absence of transcriptional activation.

Further, we examined whether NAD^+^ depletion triggers NLRP3-dependent inflammasome activation using *Nlrp3*-deficient BMDMs. FK866 along with ATP or nigericin treatment led to the robust cleavage of caspase-1 and GSDMD in wild-type BMDMs. However, FK866-mediated inflammasome activation was clearly abrogated in *Nlrp3*-knockout cells (**Figures 2C, D**). The assembly of NLRP3 inflammasome was measured by the oligomerization of ASC, an essential adaptor molecule of inflammasome, and NLRP3. FK866 + nigericin treatment induced the oligomerization of ASC in wild-type but not in *Nlrp3*-deficient BMDMs (**Figure 2E**). Furthermore, FK866 priming followed by nigericin treatment led to robust formation of speck-like NLRP3 aggregates in BMDMs expressing NLRP3-GFP (**Figure 2F**). Collectively, these results demonstrate that NAD^+^ depletion, followed by NLRP3-activating stimulation, promotes the assembly and activation of NLRP3 inflammasome.

### NAD^+^ Supplementation by NMN Abrogates FK866-Mediated NLRP3 Inflammasome Activation

**Figure 3:**
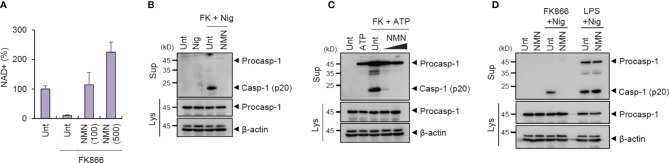
Restoration of NAD^+^ abolishes NLRP3-dependent inflammasome activation. **(A)** Quantification of intracellular NAD^+^ level in mouse BMDMs treated with FK866 and NMN (100 or 500 μM) for 21 h, as indicated (*n* = 3). **(B)** Immunoblots from mouse BMDMs treated with FK866 and NMN (500 μM) for 21 h, followed by nigericin treatment (5 μM, 1 h). **(C)** Immunoblots from mouse BMDMs treated with FK866 and NMN (500 or 1000 μM) for 21 h, followed by ATP treatment (3 mM, 1 h). **(D)** Immunoblots from mouse BMDMs treated with FK866 and NMN (500 μM) for 21 h or LPS and NMN for 3 h, followed by nigericin treatment (5 μM, 1 h). Cell culture supernatants (Sup) or cell lysates (Lys) were immunoblotted with the indicated antibodies.

To confirm whether NAD^+^ depletion is pivotal for FK866-mediated inflammasome activation, NMN was exogenously added to BMDMs. NMN restored the intracellular NAD^+^ levels against FK866-induced NAD^+^ depletion in BMDMs (**Figure 3A**). In particular, NAD^+^ restoration clearly abolished the FK866 + ATP- or nigericin-induced caspase-1 activation (**Figures 3B, C**), respectively, indicating that intracellular NAD^+^ is critical for FK866-mediated NLRP3 inflammasome activation. However, NMN treatment did not affect LPS + nigericin-induced inflammasome activation (**Figure 3D**). These results indicate that intracellular NAD^+^ decline is critical for FK866-driven NLRP3 inflammasome activation in macrophages. Of interest, LPS treatment slightly increased intracellular NAD^+^ level in BMDMs (**Supplementary Figure 3**). This result suggest that LPS priming does not mediate NAD^+^ depletion for NLRP3 activation.

### Intracellular NAD^+^ Depletion Drives Mitochondrial Translocation to Perinuclear Region

To address the non-transcriptional priming role of FK866 treatment, we assessed the involvement of SIRT1 in FK866-induced NLRP3 activation using myeloid-specific *Sirt1*-deficient BMDMs. NAD^+^ depletion results in the decrease in SIRT1 activity. Therefore, we checked whether SIRT1 inactivation can act as a priming signal for NLRP3 activation. However, FK866-mediated caspase-1 activation remained unchanged regardless of SIRT1 expression (**Supplementary Figure 4**). Furthermore, ATP alone was not able to induce caspase-1 activation in *Sirt1*-deficient BMDMs (**Supplementary Figure 4**). These findings indicate that SIRT1 is not involved in the FK866-induced inflammasome activation.

**Figure 4:**
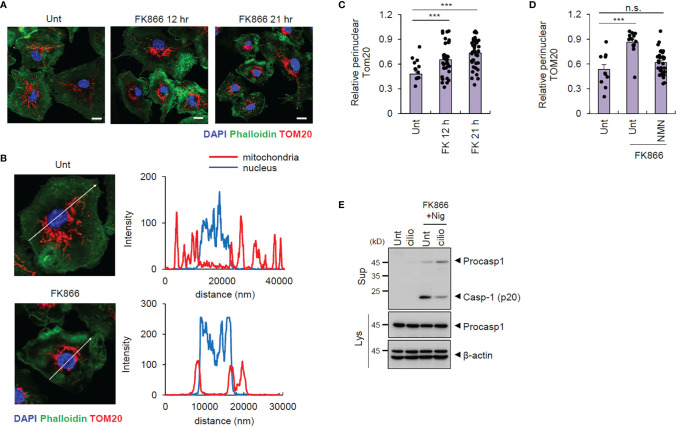
FK866-induced NAD^+^ depletion triggers mitochondrial transport to perinuclear regions. **(A)** Representative immunofluorescence images from mouse BMDMs treated with FK866 for 12 or 21 h, after staining with anti-TOM20 antibody (red) and phalloidin-Alexa 488 (green). DAPI represents the nuclear signal (blue). **(B)** Intensity profiles (right panel) of the nucleus and mitochondria along the white line (left panel) crossing the nucleus in mouse BMDMs, untreated or treated with FK866 (21 h). **(C)** Quantification of mitochondrial area surrounding the nucleus (5 μm) per total mitochondrial area, as stained by TOM20, in mouse BMDMs treated with FK866 (*n* = 22–40). **(D)** Quantification of perinuclear mitochondrial area per total mitochondrial area from mouse BMDMs treated with FK866 in the presence of NMN (500 μM) for 21 h (*n* = 11–31). **(E)** Immunoblots of mouse BMDMs pretreated with ciliobrevin D (5 μM) and FK866 (100 nM) for 12 h, followed by nigericin treatment (5 μM, 1 h). Cell culture supernatants (Sup) or cell lysates (Lys) were immunoblotted with the indicated antibodies. ***P < 0.001, n.s., not significant.

NLRP3-activating signals are known to induce mitochondrial retrograde transport into the perinuclear region to form mitochondria-associated membrane (MAM), facilitating NLRP3-ASC association to form NLRP3 inflammasome inside MAM (21, 22). Intriguingly, we observed that FK866 treatment resulted in mitochondrial translocation into the perinuclear regions (**Figures 4A–C**). Furthermore, NMN supplementation blocked the FK866-induced mitochondrial translocation (**Figure 4D** and **Supplementary Figure 5**). Additionally, the blockade of mitochondrial retrograde transport by ciliobrevin D, a selective inhibitor of dynein, abolished FK866/nigericin-induced caspase-1 activation (**Figure 4E**). These data indicate that intracellular NAD^+^ depletion drives mitochondrial translocation into the perinuclear region associated with the activation of NLRP3.

### FK866-Mediated NAD^+^ Depletion Promotes *In Vivo* NLRP3 Inflammasome Activation

**Figure 5:**
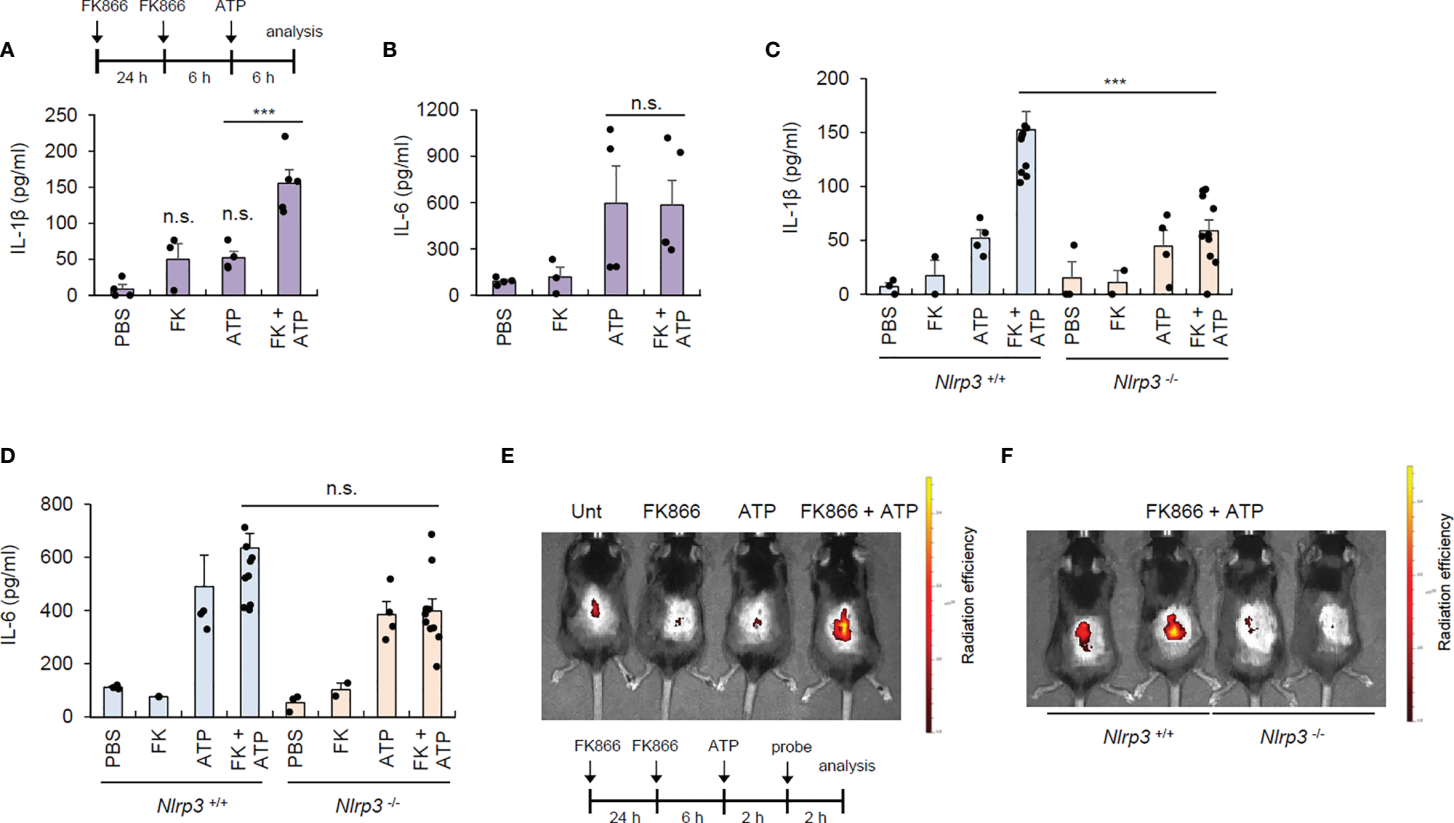
FK866-induced NAD^+^ depletion with ATP costimulation promotes NLRP3-mediated inflammasome activation *in vivo.* **(A, B)** Quantification of IL-1β **(A)** or IL-6 **(B)** in skin tissues from mouse intradermally injected with vehicle or FK866, once a day for 2 consecutive days, and then injected with ATP for 6 h (*n* = 4–5). **(C, D)** Quantification of IL-1β **(C)** or IL-6 **(D)** in skin tissues from *Nlrp3 ^+/+^* or *Nlrp3 ^-/-^* mice intradermally injected with PBS, ATP, or FK866 + ATP. (*n* = 2–10). **(E)** Representative *in vivo* fluorescence images of mice injected with PBS, ATP, or FK866 + ATP after intravenous injection with active caspase-1-specific probe. **(F)** Representative *in vivo* fluorescence images of *Nlrp3^+/+^* or *Nlrp3^-/-^* mice injected with FK866 + ATP after intravenous injection with active caspase-1-specific probe. FK866, 7 mg/kg; ATP, 125 mg/kg. ***P < 0.001, n.s., not significant.

To validate the contribution of NAD^+^ depletion in NLRP3 activation under physiological conditions, we induced topical NAD^+^ depletion in mouse skin by intradermally injecting FK866. FK866 caused negligible IL-1β production in the skin, but in the presence of ATP costimulation, FK866 induced robust IL-1β production in the skin (**Figure 5A**). However, there were no differences in IL-6 production in the lesional skin between the ATP alone and FK866-ATP-administered groups (**Figure 5B**). These data indicate that FK866 with ATP costimulation can trigger inflammasome activation in the skin. Meanwhile, ATP injection appears to promote IL-6 production in the skin possibly *via* inducing cell death. To examine whether the increased IL-1β in the skin of FK866/ATP-injected mice is due to NLRP3 inflammasome activation, we examined cytokine production in *Nlrp3*-deficient mice. Consequently, FK866 + ATP-mediated IL-1β production was markedly abolished in the skin of *Nlrp3*-knockout mice, but IL-6 production was similar between the two mice groups (**Figures 5C, D**).

To establish the *in vivo* relevance of FK866-induced inflammasome activation, we employed a selective caspase-1-activatable probe, which emits Cy5.5 fluorescence only in active caspase-1-containing cells (19). Consistent with the above data, FK866/ATP intradermal administration, but not FK866 nor ATP alone, caused robust caspase-1 activation in the mouse skin (**Figure 5E**). However, this FK866-mediated skin caspase-1 activation was not observed in the *Nlrp3*-deficient mice (**Figure 5F**), indicating that NAD^+^ depletion facilitates *in vivo* NLRP3-dependent inflammasome activation under ATP costimulation.

## Discussion

NAD^+^ decline is considered an important trigger for aging-associated pathophysiology (14). Decreased mRNA and protein level of NAMPT is potentially implicated in the aging-associated NAD^+^ decline (12, 13). Here, we employed FK866, a highly specific noncompetitive inhibitor of NAMPT, to deplete intracellular NAD^+^ and examined the role of NAD^+^ decline on the PRR-mediated response in macrophages.

Previous studies suggest that NAD^+^ homeostasis is closely related to the immune potential and polarization status of macrophages (23). In particular, they showed that FK866 treatment impairs inflammatory macrophage polarization (16), attenuates LPS-induced TLR4 signaling in human primary monocytes (15), and reduces phagocytic activity of macrophages (24), suggesting that intracellular NAD^+^ depletion might reduce the proinflammatory potential of macrophages. In contrast, results from our study indicate that NAD^+^ depletion did not significantly impair TLR4 or RIG-I signaling responses in BMDMs. We infer that the role of NAD^+^ depletion in PRR-mediated immune responses may be different depending on the cellular context. More importantly, we found that NAD^+^ depletion acts a robust non-transcriptional priming signal for NLRP3 inflammasome activation in the presence of ATP or nigericin costimulation.

NLRP3 inflammasome is unique among PRRs, as it can sense endogenous damage-associated molecular patterns (DAMPs) that cause sterile inflammation (25, 26). We thus speculate that NLRP3 inflammasome can be activated by aging-related factors or alterations. In particular, aging-associated systemic TNF-α upregulation primes NLRP3 inflammasome activation by upregulating NLRP3 expression (27). Further, macrophages from aged mice showed stronger NLRP3 inflammasome activation than from younger mice (28). However, the effect of aging-associated intracellular NAD^+^ decline on NLRP3 inflammasome activation has remained elusive.

Although we presented diverse *in vitro* and *in vivo* evidences for the NLRP3-stimulating role of NAD^+^ depletion in this study, the molecular mechanism of NAD^+^ depletion-mediated NLRP3 priming is still unclear. A potential explanation observed in this study is the mitochondrial relocation mediated by FK866. Mitochondria have been considered a hub organelle to modulate innate immune responses including RLR-mediated anti-viral signaling band NLRP3 inflammasome pathway (29). Misawa et al. showed that NLRP3-activating inducers such as ATP and nigericin lowered intracellular NAD^+^, which led to α-tubulin acetylation *via* inhibition of NAD^+^-dependent SIRT2 activity (22). The acetylated α-tubulin facilitates mitochondrial retrograde transport, which subsequently promotes the assembly of NLRP3 inflammasome. A recent study suggests that oxidative stress induces perinuclear clustering of mitochondria in a microtubule-dependent manner (30). Similarly, we found that intracellular NAD^+^ depletion induced a robust mitochondrial perinuclear clustering. We speculate that mitochondrial stress such as NAD^+^ depletion can drive mitochondrial retrograde transport, critical for the assembly of NLRP3 inflammasome. Interestingly, He et al. recently showed that aging-associated SIRT2 deficiency caused an increased NLRP3 acetylation, which facilitates the activation of NLRP3 inflammasome (28). Further investigations are needed to clarify how NAD^+^ decline can drive NLRP3 inflammasome activation under diverse circumstances.

Generally, NLRP3 inflammasome activation requires two independent priming and activating events (31). However, distinguishing the two events can be challenging. Our data demonstrated that intracellular NAD^+^ decline can provide a priming signal without transcriptional induction, and cause mitochondrial retrograde transport leading to NLRP3 activation. As mentioned earlier, NLRP3-activating signals drive mitochondrial translocation into the perinuclear region. We thus reasoned that priming events cannot be strictly distinguished from activating events under a certain context.

Danger signals or DAMPs, such as ATP, may be accumulated or present at higher levels in the aged tissue than in the young tissue (32). Therefore, chronic inflammasome activation is more likely to occur in the aged tissue with lower NAD^+^ and ATP-rich conditions. Extracellular ATP can be increased by exposure to various environmental stimuli such as ultraviolet radiation (33). Therefore, we speculate that aging-associated NAD^+^-depleted cells or tissues are more susceptible to the sudden increase in extracellular ATP. Similarly to other NLRP3-activating stimulators or alterations, we propose that aging-associated NAD^+^ decline can trigger NLRP3 inflammasome activation as a sterile inflammation in the context of ATP-rich conditions.

## Data Availability Statement

The original contributions presented in the study are included in the article/**Supplementary Material**. Further inquiries can be directed to the corresponding author.

## Ethics Statement

The animal study was reviewed and approved by Institutional Ethical Committee, Yonsei University College of Medicine.

## Author Contributions

D-WS, H-JC, and J-WY conceived and designed the study. D-WS, H-JC, and IH performed the experiments. T-YJ and H-SK generated myeloid-specific *Sirt1*-deficient mice. JHR generated caspase-1-activatable probe. J-WY supervised the entire project. D-WS, H-JC, and J-WY wrote the manuscript. All authors reviewed and approved the submitted version.

## Funding

This work was supported by the National Research Foundation of Korea Grant funded by the Korean Government (2020R1A2B5B02001823, 2020R1A4A1019009, 2019R1I1A1A01060316, 2021R1I1A1A01055624).

## Conflict of Interest

The authors declare that the research was conducted in the absence of any commercial or financial relationships that could be construed as a potential conflict of interest.

## Publisher’s Note

All claims expressed in this article are solely those of the authors and do not necessarily represent those of their affiliated organizations, or those of the publisher, the editors and the reviewers. Any product that may be evaluated in this article, or claim that may be made by its manufacturer, is not guaranteed or endorsed by the publisher.
