# Supplementary material for: Intracellular NAD+ Depletion Confers a Priming Signal for NLRP3 Inflammasome Activation
Source: Front Immunol. 2021 Dec 20;12:765477. doi: 10.3389/fimmu.2021.765477 (PMC8722528; doi:10.3389/fimmu.2021.765477)
Supplement: Supplementary file 1 [file DataSheet_1.pdf]

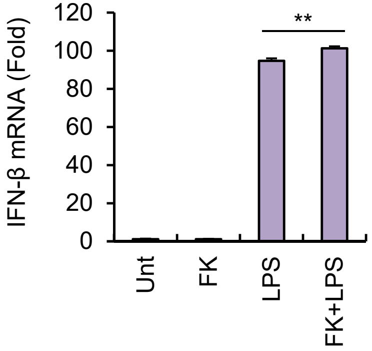

Supplementary Figure 1. NAD$^+$ depletion does not impair LPS-triggered IFN-β production. Quantification of IFN-β mRNA levels in mouse BMDMs treated with FK866 (100 nM, 20 h) or LPS treatment (0.1 µg/ml, 3 h) (n = 3). $^{**}P$ < 0.01.

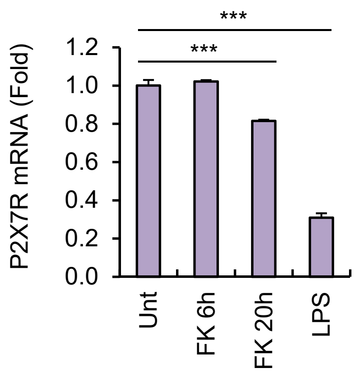

Supplementary Figure 2. NAD+ depletion does not increase P2X7 mRNA level. Quantification of P2X7 mRNA levels in mouse BMDMs treated with FK866 (100 nM, 6 or 20 h) or LPS (0.1 μg/ml, 3 h) (n = 3). ****P* < 0.001.

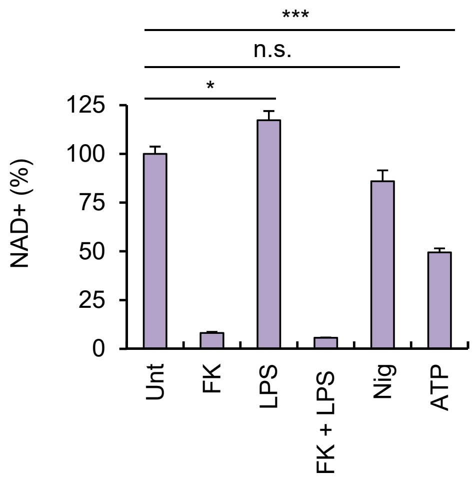

Supplementary Figure 3. Quantification of NAD^+ level in BMDMs treated with inflammasome stimulators. Quantification of NAD^+ levels in mouse BMDMs treated with FK866 (100 nM, 20 h), LPS (0.1 μg/ml, 3 h), nigericin (5 μM, 1 h) or ATP (3 mM, 1 h) (*n* = 3). **P* < 0.001, ****P* < 0.001, n.s. not significant.

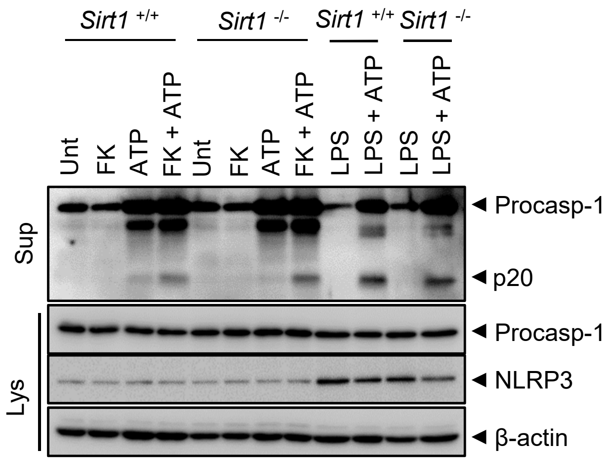

Supplementary Figure 4. SIRT1 deficiency does not provide priming signal for NLRP3 activation. (A) Immunoblots of wild-type (*Sirt1*$^{+/+}$) or myeloid-specific *Sirt1*-deficient (*Sirt1*$^{-/-}$) BMDMs treated with FK866 (100 nM, 21 h) alone, ATP (3 mM, 0.5 h) alone or FK866, followed by ATP. Cell culture supernatants (Sup) or cell lysates (Lys) were immunoblotted with the indicated antibodies.

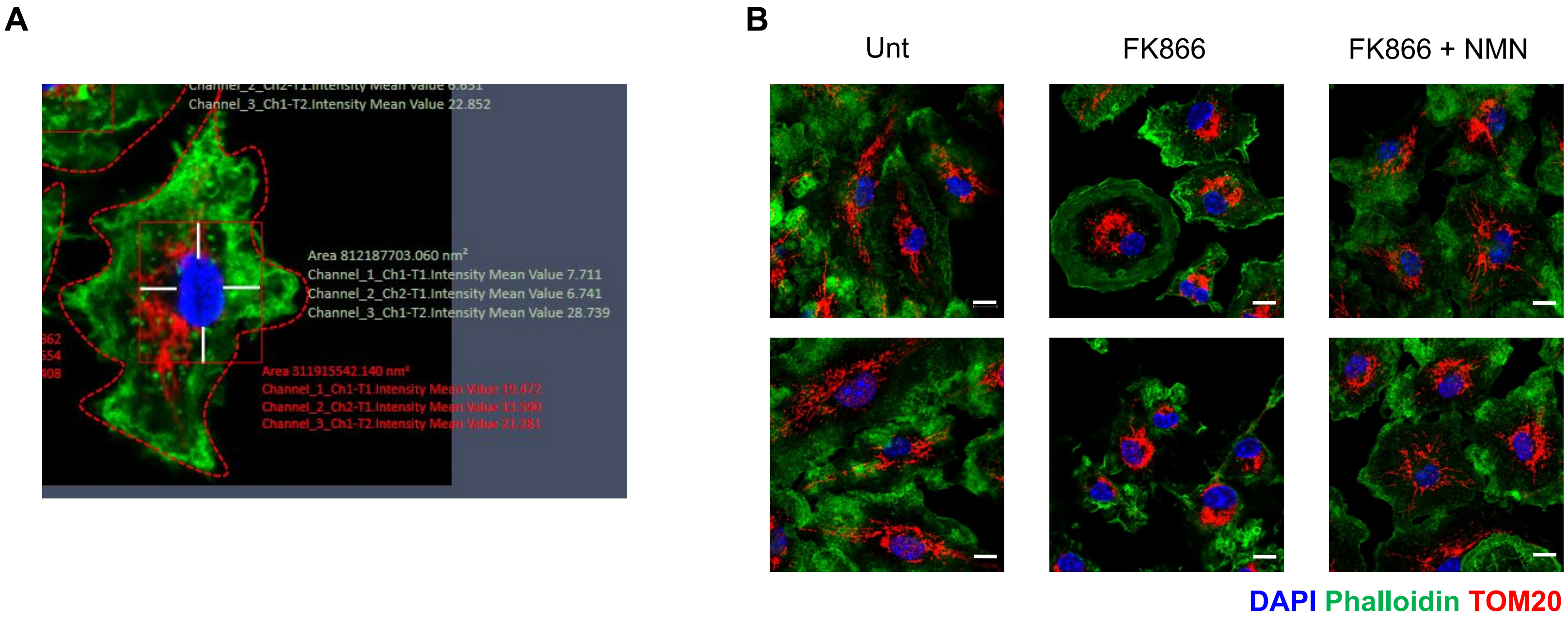

Supplementary Figure 5. FK866-induced NAD⁺ depletion drives mitochondrial transport into perinuclear region. (A) Representative immunofluorescence images from mouse BMDMs after staining with anti-Tom20 antibody (red) and phalloidin-Alexa 488 (green). Indication of perinuclear region and total intracellular area. (B) Representative immunofluorescence images from mouse BMDMs treated with FK866 (100 nM) in the presence or absence of NMN (500 μM) for 20 h, after staining with anti-Tom20 antibody (red) and phalloidin-Alexa 488 (green). DAPI represents the nuclear signal (blue).
